# 2-Methyl-6-(4-aminophenyl)-4,5-dihydro-3(2*H*)-pyridazinone Synthon for Some New Annelated 1,2,3-Selena/Thiadiazoles and 2*H*-Diazaphospholes with Anticipated Biological Activity and Quantum Chemical Calculations

**DOI:** 10.3390/molecules28031280

**Published:** 2023-01-28

**Authors:** I. E. El-Shamy, E. Hleli, M. A. El-Hashash, I. Kelnar, A. M. Abdel-Mohsen

**Affiliations:** 1 Chemistry Department, Faculty of Science, Fayoum University, Fayoum 63514, Egypt; 2 Institute of Macromolecular Chemistry, Czech Academy of Sciences, Heyrovského nám. 2, 16206 Prague, Czech Republic; 3 Chemistry Department, Faculty of Science, Ain Shams University, Cairo 11221, Egypt; 4 CEITEC, Brno University of Technology, Purkyňova 123, 61200 Brno, Czech Republic

**Keywords:** selenadiazole, thiadiazole, pyridazinone, antimicrobial activity, cytotoxicity, DFT calculations

## Abstract

A convenient and efficient synthetic protocol for the new selenadiazole. Thiadiazole and diazaphosphole derivatives incorporating a pyridazine moiety originating from 4-(4-aminophenyl)-4-oxobutanoic acid (**1**) were described. All newly synthesized compounds were evaluated for their antimicrobial activity using the disk diffusion method, and their cytotoxicity was evaluated against brine shrimp lethality bioassay. Using density functional theory (DFT), the frontier molecular orbital (FMO) and molecular electrostatic potential (MEPS) were studied to estimate the chemical reactivity and kinetic stability of each structure. Therefore, global descriptor parameters like electronegativity (χ), chemical hardness (η), and global softness (σ) were calculated. Consequently, the attained results were compared with the experimental data of the biological activity of the studied structures.

## 1. Introduction

Pyridazines and their fused heterocyclic derivatives have received significant consideration because of their synthetic and effective biological importance. Pyridazines have been reported to possess antimicrobial [1,2,3,4], antioxidant [5,6,7,8], anti-inflammatory [9,10,11,12], anticancer [8], antihypertensive [8], herbicidal activities [11] and protein tyrosine phosphatase 1B (PTP1B) inhibitors [11]. On the other hand, the interesting biological activities of *Se*-containing heterocycles are well known [11,12,13,14], including anti-inflammatory agents, immunomodifiers, cytokine inducers, and enzyme inhibitors.

Their chemotherapeutic activity has been reviewed [15]. Selenium is of essential importance to human health. It is an essential component of several major metabolic pathways, including antioxidant defense systems, thyroid hormone metabolism, and immune function. Furthermore, selenium supplementation could reduce the incidence of various types of cancers, such as prostate, lung, colon, and liver cancers [16,17,18,19,20]. It is well known that several heterocycles containing nitrogen and sulfur showed a wide variety of pharmacological and biological activities [18,19,20]. In addition, the diazole system is found in numerous antiparasitic, fungicidal, and anti-inflammatory drugs [21]. Some 1,2,3-selena, 1,3,4-(thia) diazoles, and diazaphospholes, are known to possess antitumor activity [22,23,24]. In view of these reports, we herein continue our work on biologically active nitrogen, sulfur, and selenium heterocycles [25,26]. 

We report here the synthesis of some new pyridazine scaffold incorporated 1,2,3-selena and 1,3,4-(thia) diazoles with the objective of obtaining new biologically active compounds. Here, we aim to improve the biological properties of the synthesized compounds by combining pyridazine derivatives with selenium derivative compounds. 

Furthermore, theoretical modeling was employed to discuss the electronic and chemical reactivity properties of the synthesized material. Computational calculations were performed using density functional theory (DFT) [27] using the B3LYP (Becke three-parameter Lee–Yang–Parr) exchange-correlation functional with the base set at 6-311G (d. p). Electronic properties such as HOMO and LUMO energies were also reported and discussed in view of the results of the experimental biological activities as reported in some previous research [27,28,29]. The aim of this study is to synthesize some new derivatives of annelated 1,2,3-selena/thiadiazole and 2H-diazaphospholes and investigate the biological properties (antimicrobial and biocompatibility) of the different types of bacteria and fungi of synthesized compounds. The chemical reactivity and kinetic stability of the synthesized compounds are calculated and discussed.

## 2. Results and Discussion

### 2.1. Chemistry

**Scheme 1:**
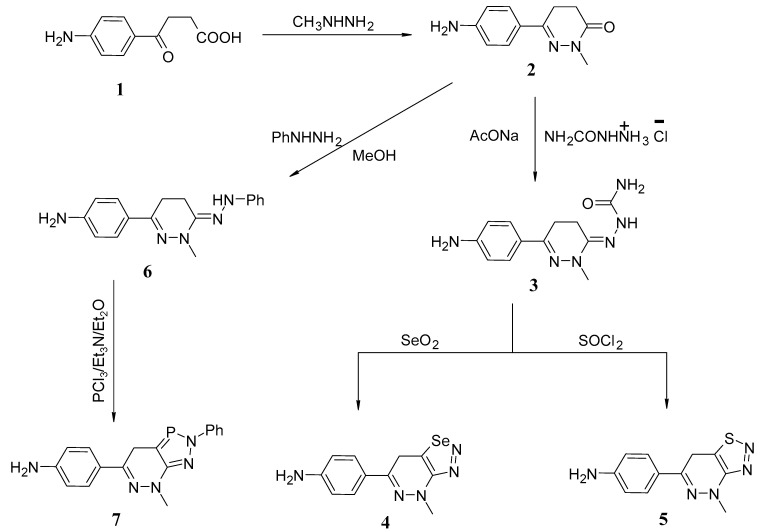
Synthesis of annelated 1,2,3-selena/thiadiazoles and 2*H*-diazaphospholes **4**, **5** and **7**.

2-Methyl-6-(4-aminophenyl)-4.5-dihydro-3(2*H*)-pyridazinone (**2**) required as starting material was readily obtained in high yields by refluxing the γ-keto acid **1** with methyl hydrazine in boiling ethanol. The melting points and spectral data of **2** agree with the reported data (mp 261–262 °C). The reaction of pyridazinone **2** with semicarbazide hydrochloride in the presence of AcONa gave the corresponding semicarbazones **3**. The IR spectra of compound **3** showed characteristic absorption bands at 1632 and 3221 cm*^−^*^1^ corresponding to the C=N and NH groups, respectively. The ^1^H-NMR spectrum of compound **3** showed pyridazinone protons C_4_ and C5 at 1.72 and 3.73 ppm, respectively. Oxidative cyclization of **3** with selenium dioxide in glacial acetic acid gave the corresponding selenadiazolopyridazine derivative **4**. However, the Hurd–Mori reaction process of **3** with excess thionyl chloride in dichloromethane at 0 °C produced the derivative **5**. The structure of compounds **4** and **5** was confirmed on the basis of their elemental analysis and spectral data. The infrared spectrum revealed that there was no absorption for the NH and CO groups. The ^1^H-NMR spectrum of compounds **4** and **5** displayed CH_2_ protons as a singlet signal at δ 1.92 and 2.24, respectively. The mass spectrum of **4** exhibited the peak molecular ion at *m/z* 292. The reaction of pyridazinone **2** with phenylhydrazine in boiling methanol produced the phenylhydrazone derivative **6**. Cyclocondensation of **6** with phosphorus trichloride in the presence of triethylamine yielded the corresponding diazaphospholopyridazin derivative **7** (Scheme 1).

**Scheme 2:**
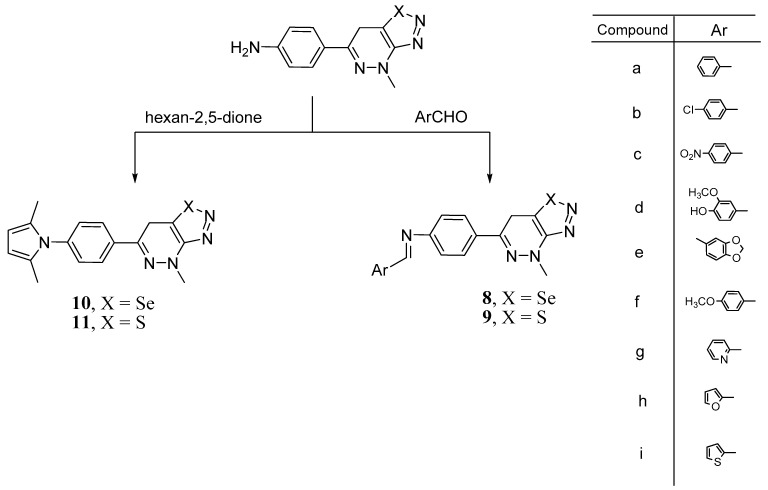
Synthesis of annelated 1,2,3-selena/thiadiazoles **8–11**.

To obtain a new series of expected biologically active Schiff bases, it was of interest to condense compounds **4** and **5** with different aromatic aldehydes, namely benzaldehyde, *p*-chlorobenzaldehyde, *p*-nitrobenazldehyde, vaniline, pipronal, anisaldehyde, pyridine-2-caboxaldehyde, furfural, and thiophene-2-caboxaldehyde, in boiling ethanol to give the corresponding Schiff bases **8a**–**i** and **9a**–**i**, respectively (Scheme 2). The IR spectrum of compounds **8a**–**i** and **9a**–**i** showed a characteristic absorption band at 1614–1629 cm^−1^ assigned for the C=N group, respectively. The ^1^H-NMR spectra of compounds **8a–i** and **9a–i** showed the presence of azomethin (CH=N) at *δ* 8.44 and 9.06 ppm, respectively. Cyclocondensation of compounds **4** and **5** with hexan-2.5-dione gave the corresponding pyrrole derivatives **10** and **11**, respectively. The IR spectra of **10** and **11** revealed no absorption for NH_2_. The ^1^H-NMR spectrum of compounds **10** and **11** showed a CH signal of the pyrrole ring at 5.22 and 5.34 ppm, respectively.

### 2.2. Biological Activities

The synthesized compounds **4**, **5**, and **8**–**11** were tested for their antimicrobial activity against *Escherichia coli*, *Staphylococcus aureus*, *Bacillus subtilis*, *Salmonella typhi* bacterial strains and *Aspergillums niger* and *Candida albicans* fungal strains using a disk diffusion assay [16,25]. Amoxicillin and ketoconazole were used as reference standards for antibacterial and antifungal activity, respectively. The preliminary screening of synthesized compounds and reference drugs was performed at fixed concentrations of 500 μg/mL. The inhibition zones of microbial growth produced by different compounds were measured at the end of an incubation period of 24 h for bacteria and 72 h for fungi. Depending on the inhibition zone, the minimum inhibitory concentration (MIC) of compounds **4**–**7** against all bacterial and fungal strains was measured by the liquid dilution method. The test compounds were dissolved in dimethylsulfoxide (DMSO) at a concentration of 500, 250, 200, 100, 50, 25, and 12.5 μg mL*^−^*^1^. The solutions of the reference drugs, amoxicillin and ketoconazole, were prepared in the same concentrations. The inoculums of the bacterial and fungal cultures were also prepared. To a series of tubes containing 1 mL of each synthesized compound solution with different concentrations, 0.2 mL of the inoculums were added. Additionally, 3.8 mL of sterile water was added to each of the test tubes. These test tubes were incubated for 24 h at 37 °C, and turbidity was observed. This method was repeated by changing the phthalazine compounds with the standard drugs amoxicillin and ketoconazole for comparison. The MIC at which no growth was observed was taken as the MIC value. A comparison of the MIC (in μg/mL) of potent compounds and standard drugs against tested strains is presented in Table 1.

All of the compounds tested exhibited different degrees of antifungal and antibacterial activities. Investigation of antibacterial screening data showed that some of the compounds were active against four pathogenic bacteria. Schiff bases **8b**, **c**, **e**, **g**, and **i** exhibited good activity against *S. aureus.* Similarly, Schiff bases **8b**, **c**, **e**, **and i** exhibited good activity against *B. subtilis.* Schiff bases **8b**, **d**, **e**, **i**, and **9b** exhibited good activity against *S. typhi*. Schiff bases **8a**, **d**, **and f** exhibited good activity against *E. coli.* The antifungal results (Table) revealed that the synthesized compounds showed variable degrees of inhibition against the tested fungi. Schiff bases **8b**, **e**, **i had** good antifungal activity against *A. niger* and *C. albicans.* From the results, it was concluded that the Schiff bases of the selenadiazolopyridazine derivative **4** showed high antimicrobial activity.

### 2.3. Cytotoxicity

Compounds **4**, **8**, and **9** were also studied for their toxicity properties using a brine shrimp lethality bioassay. The IC_50_ values (g/ml) of the compounds against the brine shrimp lethality bioassay are shown in Table 2. The biological study indicated that compound **9i** possessed the highest cytotoxicity, with a value of approximately 30 µg/mL, whereas compound **8e** exhibited the lowest cytotoxicity, with a value of approximately 330 µg/mL, against the brine shrimp lethality bioassay (Table 2). 

## 3. Chemical Reactivity Descriptors

Reactivity in chemistry is a crucial parameter since it is closely related to reaction mechanisms, allowing the understanding of chemical reactions and the improvement of the synthesis procedures for obtaining new materials. 

### 3.1. Frontier Molecular Orbital (FMO)

Frontier molecular orbital theory was hypothesized in 1952 by Kenichi Fukui, who defined the interaction of the highest molecular orbital (HOMO) and lowest unoccupied molecular orbital (LUMO) based on molecular orbital theory [30,31]. The HOMO and LUMO orbitals play a relevant role in the qualitative investigation of chemical reactivity [32]. The kinetic stability and chemical reactivity of the molecule can be characterized by computing the LUMO-HOMO energy gap. A molecule with a low LUMO-HOMO energy gap is associated with high chemical reactivity, and it would be kinetically less stable. In contrast, the high level of the LUMO-HOMO energy gap indicates that the molecule has low chemical reactivity and high kinetic stability [33,34,35]. The HOMO, LUMO energy and LUMO-HOMO energy gaps of the compounds are given in Table 3.

Here, the LUMO-HOMO energy gap is defined mathematically by Equation (1) [31].

(1) $$∆E=E_{\mathrm{LUMO}}-E_{\mathrm{HOMO}\;}$$

As exposed in Table 3, the calculations of LUMO-HOMO energy gaps using the (DFT/6-31G+) method reveal that compound **1** has the highest Eg of approximately 4.86 eV. Compound **8c** has the lowest band gap Eg in the order of about 2.56 eV. Thus, compound **1** has the lowest chemical reactivity and highest kinetic stability, and compound **8c** has the highest chemical reactivity and lowest kinetic stability. This notable gap energy difference is attributed to the difference in the structure of each compound. However, in case of varying the group function of the same compound like molecule 8, slight differences are noticed as well:

 Eg 8c (2.56 eV) < Eg 8g (3.07 eV) < Eg 8e (3.24 eV) <Eg 8i (3.26 eV)<Eg 8b (3.29 eV) < Eg 8h (3.32 eV) < Eg 8d (3.35 eV) < Eg 8a (3.36 eV) 

**Scheme 3:**
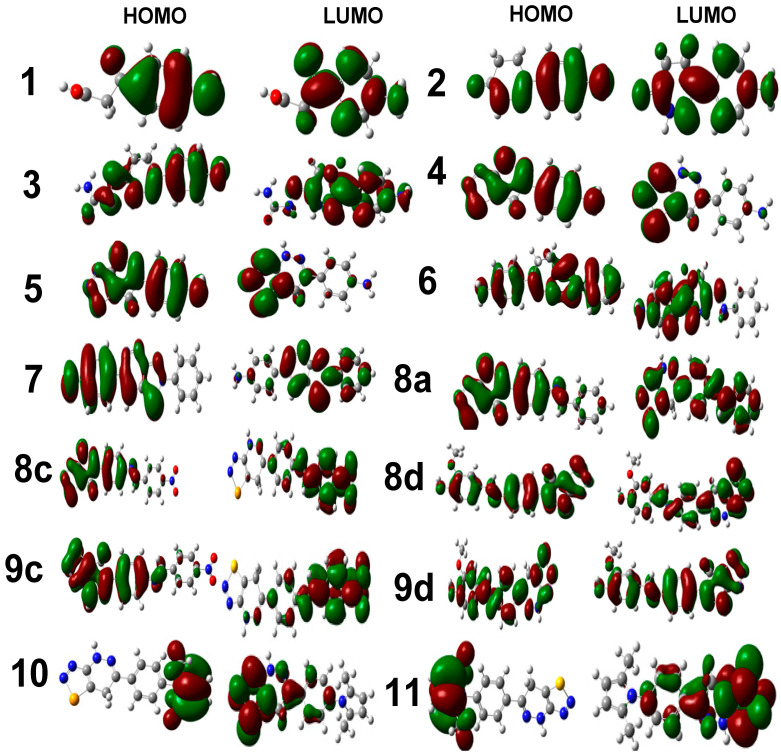
3D plots of the molecular orbitals of HOMO and LUMO of compounds calculated using the DFT (B3LYP)/6-31G+(d) method.

In addition, the substitution of the selenium (Se) atom in the 8 derivative compounds by sulfur (S) to obtain the 9 derivative compounds affected the band gap energy, which decreased, resulting in an increase in chemical reactivity. These observations highlight the importance of the structures of molecules in chemical reactivity. Thus. The theoretical calculation agrees well with the biological activity of the result of the experiment. The optimized geometric structures and 3D plots of the molecular orbitals HOMO and LUMO of all compounds are presented in Scheme 3. Therefore. It is noticed from Scheme 3 and Table S1 that for HOMO and LUMO, both are delocalized along the molecular backbone.

### 3.2. Other Global Descriptors Parameters

The study of the total reactivity of molecules is based on the calculation of global indices assumed from electronic properties. Therefore, the electronegativity (χ), global hardness (η), softness (σ), and global electrophilicity index (ω) were calculated using Equations (2)–(5) [31,36,37] are stated in Table 4.

(2) $$\chi =-1/2\left(E_{\mathrm{HOMO}}+E_{\mathrm{LUMO}}\right)$$

(3) η=(I−A)/ 2

(4) $$\omega =\frac{\chi^{2}}{2\eta }$$

(5) $$\sigma =\frac{1}{\eta }$$

In a given series of molecules, once the HOMO-LUMO gap is large, η is high, so the molecule is called hard. However, when the HOMO-LUMO is small σ is large, the molecule is subsequently called soft [30]. As mentioned in [31], chemical hardness is the opposition to a change in the density of electron clouds or the electron distribution of a chemical system, and chemical softness is the inverse of chemical hardness. This means that a molecule with low gap energy has the highest softness and the highest electronegativity. In Table 4 are listed the calculated molecular properties of the structures studied. 

From Table 4, it was noticed that compound 8c has the highest overall electrophilicity index (ω), highest softness (σ), and the highest electronegativity (χ), which is related to the lowest HOMO-LUMO gap energy. Hence it is the best electron donor based on the frontier molecular orbital (FMO) section.

### 3.3. Molecular Electrostatic Potential

**Scheme 4:**
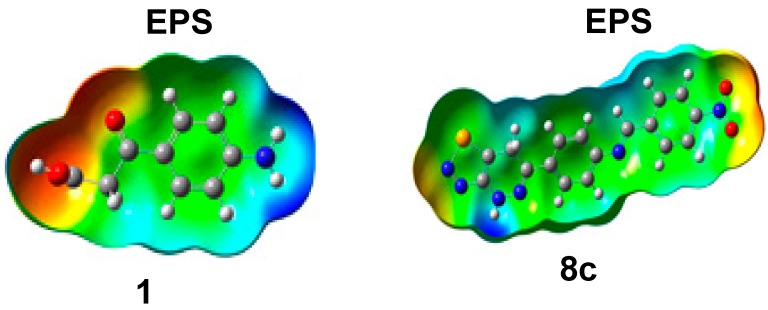
Representative molecular electrostatic potentials (MESP) mapped on the electron density surface calculated by the B3LYP/6-31+G(d) method for compounds **1** and **8b.** Symbols are O (red), C (gray), H (white), and N (blue). The electrostatic potential increases in the order red (electrophilic)< orange < yellow < green < blue (nucleophilic).

The molecular electrostatic potential (MEP) is an important parameter for predicting and understanding the molecular shape, electron density, relative polarity, and molecular interaction of electrophilic-nucleophilic sites. Thus. to predict the chemically reactive sites required for the electrophilic and nucleophilic attacks of molecules 1 and 8c. MESP plots were calculated using the B3LYP/6-31+G(d) model and the DFT method for the optimized structure already obtained (Scheme 4). In this context. Electrostatic potential values are identified by different colors on the surface; the red region is related to the lowest or most negative electrostatic potential. The blue region identifies the highest or most positive electrostatic potential, and the green region refers to the area of zero potential. Therefore, the electrostatic potential rises in the order red < orange < yellow < green < blue.

As shown in Scheme 4 for molecule 1. The negative electrostatic potential region (red and yellow) is mainly localized to the oxygen atoms. The oxygen atom of the carbonyl group is the most reactive site for an electrophilic attack. The highest positive electrostatic potential region (blue) is located on the nitrogen atom linked to hydrogen, which explains its most reactive site for a nucleophilic attack. Furthermore, for compound **8c**, it is noticed that the most electrophilic site is located over the oxygen atoms, although the most nucleophilic attack site is located on the nitrogen related to one hydrogen.

### 3.4. Dimer Study

**Scheme 5:**
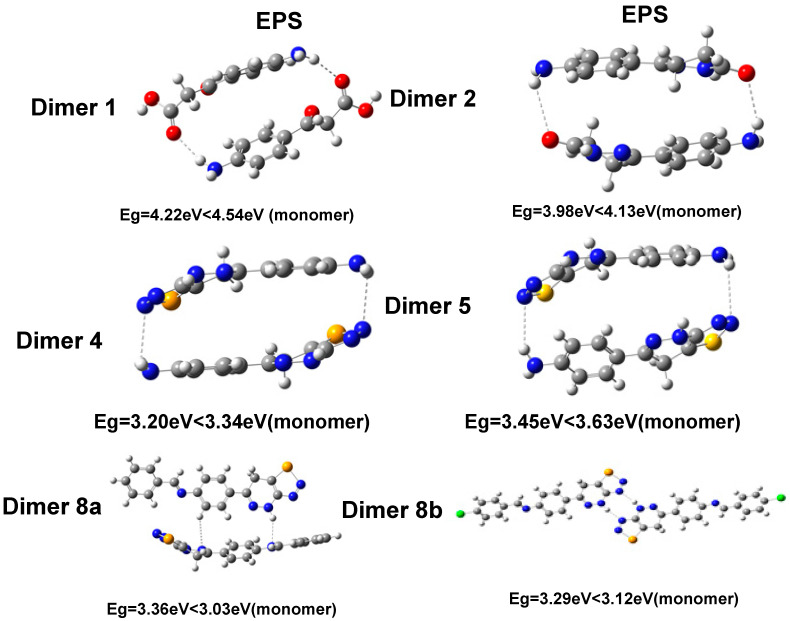
Optimized dimer structure and their energy gap.

In order to investigate the influence of chain length on the chemical reactivity of each structure and to predict the chemical reactivity of a dimer structure resulting from a physical interaction between two monomers placed antisymmetrically, the gap energy for each optimized dimer structure was determined using the DFT method. B3LYP model.6-31G+ basis set (Scheme 5).

As illustrated in Scheme 5, the gap energy value is reduced for the dimer as compared to the monomer for each structure. As an example, compound 1 Eg decreases from 4.54 eV (monomer) to 4.22 eV (dimer). This is a sign of the highest chemical reactivity and the lowest kinetic stability for a longer chain, as referred to in [38]. Moreover. in the case of component 8. selenium plays a good role in increasing the chemical reactivity compared to the other components. Thus, for the dimer of sample 8, the existence of two selenium atoms will further increase its chemical reactivity. Furthermore, this decrease in the energy gap value is due to the intermolecular interaction that can arise between the two monomers, such as hydrogen bonding interaction [39]. Thus, DFT represents a pertinent theory for predicting the chemical reactivity of the studied component.

## 4. Experimental

### 4.1. Synthesis

The melting points were determined on the Electrothermal 9100 melting point apparatus (Electrothermal, Staffordshire, UK) and were not corrected. The IR spectra (KBr) were recorded on an FT-IR NEXCES spectrophotometer (Shimadzu, Kyoto, Japan). The 1H-NMR spectra were measured with a Jeol ECA 500 MHz instrument (Tokyo, Japan) in DMSO-*d*6, and chemical changes were recorded in δ ppm relative to TMS. Mass spectra (EI) were run at 70 eV with a Finnigan SSQ 7000 spectrometer. The purity of the compounds was checked on aluminum plates coated with silica gel (Merck, Darmstadt, Germany). The elemental analysis for C, H, N and S was performed using a Costech model 4010, and the percentage values agreed with the proposed structures within ±0.4% of the theoretical values.

*2-Methyl-6-(4-aminophenyl)-4,5-dihydro-3(2H)-pyridazinone (mp 216–217 °C) 2-(6-(4-aminophenyl)-2-methyl-4,5-dihydropyridazin-3 (2H) ylidene) hydrazine carboxamide (3):* A solution of pyridazinone **2** (0.01 mol), semicarbazide hydrochloride (2.23 g, 0.02 mol), anhydrous CH_3_COONa (3 g) in absolute methanol (30 mL) and a few drops of glacial acetic acid (0.5 mL) was heated under reflux for 4 h. The completion of the reaction was monitored by TLC. The reaction mixture was cooled to room temperature and poured onto crushed ice. The precipitate formed was collected by filtration under vacuum suction and washed with cold water, dried, and recrystallized from ethanol to give compound **3** with a 75% yield, mp 261–262 °C; IR (KBr) v_max_ 3459, 3221 (NH_2_, NH), 1659 (C=O), 1632 (C=N) cm^−1^; ^1^H-NMR (DMSO-d_6_, δ): 1.72 (t, 2H, J = 8.22 Hz, CH_2_ pyridazine moiety), 3.02 (s, 3H, CH_3_), 3.73 (t, 2H, J = 8.22 Hz, CH_2_ pyridazine moiety), 6.11 (s, 2H, NH_2_ exchangeable with D_2_O ), 6.40 (s, 2H, NH_2_, exchangeable with D2O), 7.11–8.02 (m, 4H, Ar–H), 8.94 (s, broad, 1H, NH, exchangeable with D_2_O); ^13^C-NMR (DMSO-d_6_, δ): 15.5 (CH_2_), 27.1 (CH_2_), 48.3 (CH_3_), 110.2, 124.1, 131.2, 144.5, 149.0, 151.1, 160.2 (C=O); EI MS *m*/*z*: 260 [M]^+^ (29). Anal. C_12_H_16_N_6_O: C, 55.37%; H, 6.20%; N, 32.29%; Calcd for C, 55.39%; H, 6.15%; N, 32.33%.

*4-(4-Methyl-4,7-dihydro-[1,2,3]selenadiazolo [4,5-c]pyridazin-6-yl)aniline(4):* A solution of compounds **3** (0.01 mol) in glacial acetic acid (20 mL) was warmed at 70 °C with stirring, then selenium dioxide (0.01 mol) was added in parts for a period of 40 min, and stirring was continued for another 9 h. After the mixture was cooled, the reaction mixture was filtered to remove the deposited Se, and then the filtrate was poured onto crushed ice. The precipitate formed was collected by filtration under vacuum suction and washed with a cold solution of H2O / Na2CO3 and then H_2_O. The obtained product was dried and crystallized from ethanol to give compound **4** in yields: 65%. Mp 140–141 °C; IR (KBr) *v_max_* 3489, 3395 (NH_2_), 1611 (C=N) cm^−1^; ^1^H-NMR (DMSO-d_6_, *δ*): 1.92 (s, 2H, CH_2_ pyridazine moiety), 3.00 (s, 3H, CH_3_), 6.20 (s, 2H, NH_2_ exchangeable with D_2_O ), 7.09–8.03 (m, 4H, Ar–H); ^13^C-NMR (DMSO-d_6_, δ): 22.2 (CH_2_), 48.8 (CH_3_), 111.0, 129.0, 130.0, 134.1, 139.0, 144.1, 152.4; Anal. C_11_H_11_N_5_Se: C, 45.22%; H, 3.79%; N, 23.97%; Calcd. for C, 45.18%; H, 3.82%; N, 23.90%.

*4-(4-Methyl-4,7-dihydro-[1,2,3]thiadiazolo [4,5-c] pyridazin-6-yl) aniline (5): Semicarbazone* **3** (0.01 mol) was added in portions to an excess of freshly distilled thionyl chloride (10 mL) while it was cooled to 5 C with a freezing mixture. The reaction mixture was then allowed to reach room temperature. After 60 min, methylene chloride (20 mL) was added, and the resulting mixture was decomposed with saturated sodium carbonate. The methylene chloride layer was thoroughly washed with water and dried over anhydrous Na_2_SO_4_. The evaporation of the solvent gave a syrupy substance, which was purified by column chromatography to get a pure **5** in 70% yield. Mp 197–198 °C; IR (KBr) *v_max_* 3489, 3395 (NH_2_), 1611 (C=N) cm-1; ^1^H-NMR (DMSO-d_6_, *δ*): 2.24 (s, 2H, CH_2_ pyridazine moiety), 3.05 (s, 3H, CH_3_), 6.34 (s, 2H, NH_2_ exchangeable with D_2_O ), 7.02–7.94 (m, 4H, Ar–H); ^13^C-NMR (DMSO-d_6_, δ): 28.3 (CH_2_), 47.5 (CH_3_), 115.3, 126.1, 133.2, 147.1, 148.3, 149.1, 157.6; EI MS *m/z*: 245 [M]^+^ (11). Anal. C_11_H_11_N_5_S: C, 53.86%; H, 4.52%; N, 28.55%; S, 13.07%; Calcd. for C, 53.80%; H, 4.57%; N, 28.50%; S, 13.12%.

*4-(1-methyl-6-(2-phenylhydrazono)-1,4,5,6-tetrahydropyridazin-3-yl)aniline (6):* To pyradizinone **2** (0.05 mol) dissolved in methanol (20 mL), phenylhydrazine (0.05 mol) was added and refluxed for 10 h. Then the reaction mixture was concentrated and cooled. The solid was collected by filtration, washed with water, dried and recrystallized from absolute ethanol to give **6** in 60% yields. Mp 155–156 °C; IR (KBr) *v_max_* 3444, 3367(NH_2_), 3125 (NH), cm^−1^; 1H-NMR (DMSO-d6, δ): 1.70 (t, 2H, *J* = 8.22 Hz, CH_2_ pyridazine moiety), 3.11 (s, 3H, CH_3_), 3.70 (t, 2H, *J* = 8.22 Hz, CH_2_ pyridazine moiety), 6.32(s, 2H, NH_2_ exchangeable with D_2_O ), 7.02–8.23 (m, 9H, Ar–H), 9.90 (s, broad, 1H, NH, exchangeable with D_2_O); EI MS *m/z*:293 [M]^+^ (19). Anal. C_17_H_19_N_5_: C, 69.60%; H, 6.53%; N, 23.87%; Calcd. for C, 69.58%; H, 6.58%; N, 23.83%.

*4-(4-methyl-2-phenyl-4,7-dihydro-2H-[1,2,3]diazaphospholo [5,4-c]pyridazin-6-yl) aniline (7):* To a stirred solution of phosphorus trichloride (0.015 mol) and anhydrous diethyl ether (30 mL) in a nitrogen atmosphere maintained at –5 to –10 °C, phenyl hydrazone **6** (0.01mol) in dry ether (15 mL) was added dropwise. To this, triethylamine (0.012 mol) was added, and stirring was continued for 5 h. The ethereal layer was separated, washed with sodium bicarbonate solution, water, and dried (anhydrous sodium sulfate). The ether was removed under reduced pressure, and the resulting residue was purified by filtration through a silica gel column using n-hexane: ethyl acetate (1.5:1) as eluent to yield **7** in 58%. Mp 188–189 ºC; IR (KBr) *v_max_* 3430, 3388 (NH_2_) cm^−1^; 1H-NMR (DMSO-d6, δ): 1.11 (s, 2H, CH_2_ pyridazine moiety), 3.14 (s, 3H, CH_3_), 6.42 (s, 2H, NH_2_ exchangeable with D_2_O), 6.97–8.20 (m, 9H, Ar–H). Anal. C_17_H_16_N_5_P: C, 63.55%; H, 5.02%; N, 21.80%; Calcd. for C, 63.50%; H, 5.09%; N, 21.77%.

*General procedure for the synthesis of Schiff bases **8a–i** and **9a–i**:* A solution of **4** and/or **5** (0.001 mmol) and appropriate aromatic aldehyde, namely benzaldehyde, *p*-chlorobenzaldehyde, *p*-nitrobenazldehyde, vaniline, pipronal, anisaldehyde, pyridine-2-caboxaldehyde, furfural and thiophene-2-caboxaldehyde (0.001 mol) in ethanol (30 mL) was refluxed in the presence of a few drops of glacial acetic acid for 4–7 h. After being cooled, the precipitated solid was filtered off, dried, and crystallized from the appropriate solvent to give **6ai** and **7ai,** respectively.

*N-Benzylidene-4-(4-methyl-4,7-dihydro-[1,2,3]selenadiazolo [4,5-c]pyridazin-6-yl) aniline (8a):* Yield: 65%, mp 63–64 °C; IR (KBr) *v_max_* 1614 (C=N) cm^−1^; ^1^H-NMR (DMSO-d_6_, *δ*): 1.95 (s, 2H, CH_2_ pyridazine moiety), 3.01 (s, 3H, CH_3_), 6.99–7.98 (m, 9H, Ar–H), 8.77 (s, H, CH); ^13^C-NMR (DMSO-d_6_, δ): 21.5 (CH_2_), 47.9 (CH_3_), 120.1, 126.7, 127.2, 128.6, 130.0, 131.4, 133.0, 135.5, 149.4, 140.4, 149.2, and 158.8 (C=N); EI MS *m/z* 380 [M]^+^ (10). Anal. C_18_H_15_N_5_Se: C, 56.85%; H, 3.98%; N, 18.42%; Calcd. for C, 56.80%; H, 4.02%; N, 18.48%.

*N-(4-Chlorobenzylidene)-4-(4-methyl-4,7-dihydro-[1,2,3]selenadiazolo [4,5-c] pyridazin-6-yl)aniline (8b):* Yield: 60%, mp 70–71 °C; IR (KBr) *v_max_* 1618(C=N) cm^−1^; ^1^H-NMR (DMSO-d_6_, *δ*): 2.11(s, 2H, CH_2_ pyridazine moiety), 3.22 (s, 3H, CH_3_), 7.00–7.99 (m, 8H, Ar–H), 8.84 (s, H, CH); ^13^C-NMR (DMSO-d_6_, δ): 25.7 (CH_2_), 48.9 (CH_3_), 120.6, 127.7, 128.6, 129.8, 133.0, 134.5, 136.4, 137.0, 139.1, 143.2, 150.6, and 161.2 (C=N). Anal. C_18_H_14_ClN_5_Se: C, 52.13%; H, 3.40%; Cl, 8.55%; N, 16.89%; Calcd. for C, 52.18%; H, 3.45%; Cl, 8.50%; N, 17.03%.

*N-(4-nitropenzylidene)-4-(4-methyl-4,7-dihydro-[1,2,3]selenadiazolo [4,5-c] pyridazin-6-yl) aniline (8c):* Yield: 59%, mp 74–75 °C; IR (KBr) *v_max_* 1620(C=N) cm^-^1; 1H-NMR (DMSO-d_6_, *δ*): 2.03(s, 2H, CH_2_ pyridazine moiety), 3.19 (s, 3H, CH_3_), 7.07–7.90 (m, 8H, Ar–H), 8.80 (s, H, CH); 13C-NMR (DMSO-d6, δ): 26.0(CH2), 49.8(CH3), 120.2, 121.7, 125.5, 129.0, 131.1, 135.6, 139.0, 143.6, 144.8, 148.7, 151.1, 158.9 (C=N); Anal. C_18_H_14_ClN_5_Se: C, 50.83%; H, 3.32%; N, 19.76%; Calcd. for C, 50.80%; H, 3.30%; N, 19.73%.

*2-Methoxy-5-((4-(4-methyl-4,7-dihydro-[1,2,3]selenadiazolo [4,5-c]pyridazin-6-yl) phenylamino)methyl)phenol (8d):* Yield: 66%, mp 109–110 °C; IR (KBr) *v_max_* 1612(C=N) cm^−1^; ^1^H-NMR (DMSO-d_6_, *δ*): 1.90 (s, 2H, CH_2_ pyridazine moiety), 2.96 (s, 3H, CH_3_), 3.83 (s, 3H, CH_3_), 4.97 (s, 1H, OH exchangeable with D_2_O ), 7.04–7.85 (m, 7H, Ar–H), 8.74 (s, H, CH); ^13^C-NMR (DMSO-d_6_, δ): 23.2 (CH_2_), 47.7 (CH_3_), 60.1 (OCH_3_), 110.7, 115.6, 119.2, 121.0, 127.7, 129.3, 130.4, 132.2, 135.5, 139.4, 143.6, 149.8, 151.2, and 162.1; Anal. C_19_H_17_N_5_O_2_Se: C, 53.53%; H, 4.02%; N, 16.43%; Calcd. for C, 53.58%; H, 4.05%; N, 16.40%.

*N-(Benzo[d][1,3]dioxol-5-ylmethylene)-4-(4-methyl-4,7-dihydro-[1,2,3]selenadiazolo [4,5-c]pyridazin-6-yl)aniline (8e):* Yield: 69%, mp 101–102 °C; IR (KBr) *v_max_* 1610 (C=N) cm^−1^; ^1^H-NMR (DMSO-d_6_, *δ*): 2.08(s, 2H, CH_2_ pyridazine moiety), 3.00 (s, 3H, CH_3_), 6.45 (s, 2H, CH_2_ pipronal moiety), 7.05–7.89 (m, 7H, Ar–H), 8.70 (s, H, CH); ^13^C-NMR (DMSO-d_6_, δ): 25.0 (CH_2_), 49.1 (CH_3_), 100.3 (CH_2_), 109.5, 113.2, 118.6, 123.3, 127.7, 129.5, 133.1, 136.0, 139.1, 140.4, 145.9, 149.8, 153.0, and 159.3 (C=N); EI MS *m/z* 424 [M]^+^ (10). Anal. C_19_H_15_N_5_O_2_Se: C, 53.78%; H, 3.56%; N, 16.51%; Calcd. for C, 53.74%; H, 3.59%; N, 16.47%.

*N-(4-Methoxybenzylidene)-4-(4-methyl-4,7-dihydro-[1,2,3]selenadiazolo [4,5-c] pyridazin-6-yl)aniline (8f):* Yield: 60%, mp 69–70 °C; IR (KBr) *v_max_* 1614 (C=N) cm^-^1; 1H-NMR (DMSO-d_6_, *δ*): 1.91(s, 2H, CH_2_ pyridazine moiety), 3.01 (s, 3H, CH_3_), 3.92 (s, 3H, CH_3_), 7.12–7.90 (m, 8H, Ar–H), 8.76 (s, H, CH); ^13^C-NMR (DMSO-d_6_, δ): 23.5 (CH_2_), 47.8 (CH_3_), 58.7 (OCH3), 115.0, 123.2, 126.6, 127.9, 129.4, 130.1, 131.8, 137.0, 140.2, 150.3, and 161.0 (C=N), 164.2; Anal. C_19_H_17_N_5_OSe: C, 55.61%; H, 4.18%; N, 17.07%; Calcd. for C, 55.66%; H, 4.21%; N, 17.00%.

*4-(4-Methyl-4,7-dihydro-[1,2,3]selenizolo [4,5-c] pyridazin-6-yl)-N-(pyridin-2-yl methylene) aniline (8g):* Yield: 58%, mp 98–99 °C; IR (KBr) *v_max_* 1613 (C=N) cm^−1^; ^1^H-NMR (DMSO-d_6_, *δ*): 2.10 (s, 2H, CH_2_ pyridazine moiety), 3.11 (s, 3H, CH_3_), 7.10–8.55 (m, 8H, Ar–H), 8.66 (s, H, CH); ^13^C-NMR (DMSO-d_6_, δ): 21.8 (CH_2_), 48.4 (CH_3_), 120.3, 124.2, 126.9, 130.1, 134.2, 137.0, 137.8, 139.6, 140.8, 145.5, 148.3, 150.2, and 154.6 (C=N); Anal. C_17_H_14_N_6_Se: C, 53.55%; H, 3.70%; N, 22.04%; Calcd. for C, 53.59%; H, 3.66%; N, 22.09%. 

*N-(Furan-2-ylmethylene)-4-(4-methyl-4,7-dihydro-[1,2,3]selenadiazolo [4,5-c] pyridazin-6-yl) aniline (8h):* Yield: 66%, mp 83–84 °C; IR (KBr) *v_max_* 1619 (C=N) cm^−1^; ^1^H-NMR (DMSO-d_6_, *δ*): 1.96 (s, 2H, CH_2_ pyridazine moiety), 2.95 (s, 3H, CH_3_), 6.70–7.88 (m, 7H, Ar–H), 8.10 (s, H, CH); ^13^C-NMR (DMSO-d_6_, δ): 24.0 (CH_2_), 49.1 (CH_3_), 110.3, 117.2, 123.5, 127.9, 130.3, 132.0, 136.6, 140.2, 143.4, 148.1, 148.9, and 152.2 (C=N); Anal. C_16_H_13_N_5_OSe: C, 51.90%; H, 3.54%; N, 18.91%; Calcd. for C, 51.96%; H, 3.50%; N, 18.88%.

*4-(4-Methyl-4,7-dihydro-[1,2,3]selenadiazolo [4,5-c]pyridazin-6-yl)-N-(thiophen-2-yl methylene)aniline (8i):* Yield: 61%, mp 77–78 °C; IR (KBr) *v_max_* 1617 (C=N) cm^−1^; ^1^H-NMR (DMSO-d_6_, *δ*): 2.09 (s, 2H, CH_2_ pyridazine moiety), 3.04 (s, 3H, CH_3_), 6.97–7.89 (m, 7H, Ar–H), 8.80 (s, H, CH); Anal. C_16_H_13_N_5_SSe: C, 49.74%; H, 3.39%; N, 18.13%; S, 8.30%; Calcd. for C, 49.70%; H, 3.42%; N, 18.10%; S, 8.28%. 

*N-Benzylidene-4-(4-methyl-4,7-dihydro-[1,2,3]thiadiazolo [4,5-c]pyridazin-6-yl) aniline (9a):* Yield: 71%, mp 80–81 °C; IR (KBr) *v_max_* 1622(C=N) cm^−1^; ^1^H-NMR (DMSO-d_6_, *δ*): 2.19(s, 2H, CH_2_ pyridazine moiety), 3.06 (s, 3H, CH_3_), 7.09–7.90 (m, 9H, Ar–H), 8.79 (s, H, CH); ^13^C-NMR (DMSO-d_6_, δ): 29.2(CH_2_), 48.3(CH_3_), 123.7, 129.0, 129.8, 130.3, 132.4, 133.5, 137.8, 145.6, 148.4, 153.7, 157.3, 163.2(C=N); Anal. C_18_H_15_N_5_S: C, 64.84%; H, 4.53%; N, 21.01%; S, 9.62%; Calcd. for C, 64.88%; H, 4.57%; N, 20.97%; S, 9.60%.

*N-(4-Chlorobenzylidene)-4-(4-methyl-4,7-dihydro-[1,2,3]thiadiazolo [4,5-c] pyridazin-6-yl) aniline (9b):* Yield: 66%, mp 93–94 °C; IR (KBr) *v_max_* 1621 (C=N) cm^−1^; ^1^H-NMR (DMSO-d_6_, *δ*): 2.83(s, 2H, CH_2_ pyridazine moiety), 3.29 (s, 3H, CH_3_), 7.04–7.97 (m, 8H, Ar–H), 8.86 (s, H, CH); ^13^C-NMR (DMSO-d_6_, δ): 29.5(CH_2_), 48.2 (CH_3_), 120.5, 124.8, 129.1, 130.5, 131.1, 133.6, 137.0, 143.3, 145.7, 150.0, 151.2, and 161.8 (C=N); EI MS *m/z* 367 [M]^+^ (20). Anal. C_18_H_14_ClN_5_S: C, 58.77%; H, 3.84%; Cl, 9.64%; N, 19.04%; S, 8.72%; Calcd. for C, 58.79%; H, 3.80%; Cl, 9.68%; N, 19.09%; S, 8.70%. 

*N-(4-nitropenzylidene)-4-(4-methyl-4,7-dihydro-[1,2,3]thiadiazolo [4,5-c] pyridazin-6-yl) aniline (9c):* Yield: 69%, mp 99–100 °C; IR (KBr) *v_max_* 1618 (C=N) cm^−1^; ^1^H-NMR (DMSO-d_6_, *δ*): 2.70(s, 2H, CH_2_ pyridazine moiety), 3.12 (s, 3H, CH_3_), 7.02–7.85 (m, 8H, Ar–H), and 8.79 (s, H, CH); Anal. C_18_H_14_N_6_O_2_S: C, 57.13%; H, 3.73%; N, 22.21%; S, 8.47%; Calcd. for C, 57.17%; H, 3.78%; N, 22.20%; S, 8.50%. 

*2-Methoxy-5-((4-(4-methyl-4,7-dihydro-[1,2,3]thiadiazolo [4,5-c]pyridazin-6-yl) phenyl imino)methyl)phenol (9d):* Yield: 71%, mp 133–134 °C; IR (KBr) *v_max_* 1617(C=N) cm^−1^. ^1^H-NMR (DMSO-d_6_, *δ*): 2.53(s, 2H, CH_2_ pyridazine moiety), 2.99 (s, 3H, CH_3_), 3.87 (s, 3H, CH_3_), 4.99(s, 1H, OH exchangeable with D_2_O ), 7.00–7.90 (m, 7H, Ar–H), 8.79 (s, H, CH); ^13^C-NMR (DMSO-d_6_, δ): 27.0 (CH_2_), 45.7 (CH_3_), 60.3 (OCH_3_), 110.2, 113.5, 120.0, 121.5, 127.4, 128.8, 133.1, 140.4, 148.3, 150.0, 152.1, 153.3, 156.7, and 163.0 (C=N); Anal. C_19_H_17_N_5_O_2_S: C, 60.14%; H, 4.52%; N, 18.46%; S, 8.45%; Calcd. for C, 60.17%; H, 4.57%; N, 18.40%; S, 8.43%. 

*N-(Benzo[d][1,3]dioxol-5-ylmethylene)-4-(4-methyl-4,7-dihydro-[1,2,3]thiadiazolo [4,5-c]pyridazin-6-yl)aniline (9e):* Yield: 67%, mp 110–111 °C; IR (KBr) *v_max_* 1620 (C=N) cm^−1^; ^1^H-NMR (DMSO-d_6_, *δ*): 2.68 (s, 2H, CH_2_ pyridazine moiety), 3.06 (s, 3H, CH_3_), 6.48 (s, 2H, CH_2_ pipronal moiety), 7.01–7.94 (m, 7H, Ar–H), 8.73 (s, H, CH); ^13^C-NMR (DMSO-d_6_, δ): 30.2 (CH_2_), 48.8 (CH_3_), 98.9 (OCH_2_O), 107.4, 109.0, 123.3, 124.8, 130.1, 132.2, 135.7, 141.1, 145.8, 149.1, 150.8, 153.1, 156.6, and 161.8 (C=N); EI MS *m/z* 377 [M]^+^ (18). Anal. C_19_H_15_N_5_O_2_S: C, 60.46%; H, 4.01%; N, 18.56%; S, 8.50%; Calcd. for C, 60.39%; H, 4.07%; N, 18.53%; S, 8.48%. 

*N-(4-Methoxybenzylidene)-4-(4-methyl-4,7-dihydro-[1,2,3]thiadiazolo [4,5-c]pyridazin-6-yl)aniline (9f):* Yield: 69%, mp 88–89 °C; IR (KBr) *v_max_* 1623 (C=N) cm^−1^; ^1^H-NMR (DMSO-d_6_, *δ*): 2.21(s, 2H, CH_2_ pyridazine moiety), 3.02 (s, 3H, CH_3_), 3.94 (s, 3H, CH_3_), 7.10–7.92 (m, 8H, Ar–H), 8.79 (s, H, CH); ^13^C-NMR (DMSO-d_6_, δ): 25.3(CH_2_), 49.5(CH_3_), 60.2(OCH3), 111.1, 120.3, 127.7, 129.0, 133.2, 135.8, 146.2, 148.5, 150.4, 151.2, and 159.0 (C=N), 164.1; EI MS *m/z* 363 [M]^+^ (34). Anal. C_19_H_17_N_5_OS: C, 62.79%; H, 4.71%; N, 19.27%; S, 8.82%; Calcd. for C, 62.79%; H, 4.71%; N, 19.27%; S, 8.82%. 

*4-(4-Methyl-4,7-dihydro-[1,2,3]thiadiazolo [4,5-c]pyridazin-6-yl)-N-(pyridin-2-yl methylene)aniline (9g):* Yield: 72%, mp 105–106 °C; IR (KBr) *v_max_* 1622 (C=N) cm^−1^; ^1^H-NMR (DMSO-d_6_, *δ*): 2.81(s, 2H, CH_2_ pyridazine moiety), 3.13 (s, 3H, CH_3_), 7.02–8.57 (m, 8H, Ar–H), and 8.69 (s, H, CH); Anal. C_17_H_14_N_6_S: C, 61.06%; H, 4.22%; N, 25.13%; S, 9.59%; Calcd. for C, 60.98%; H, 4.20%; N, 25.17%; S, 9.61%. 

*N-(Furan-2-ylmethylene)-4-(4-methyl-4,7-dihydro-[1,2,3]thiadiazolo [4,5-c]pyridazin-6-yl) aniline (9h):* Yield: 70%, mp 97–98 °C; IR (KBr) *v_max_* 1621 (C=N) cm^−1^; ^1^H-NMR (DMSO-d_6_, *δ*): 2.55(s, 2H, CH_2_ pyridazine moiety), 3.10 (s, 3H, CH_3_), 6.68–7.89 (m, 7H, Ar–H), 8.15 (s, H, CH); ^13^C-NMR (DMSO-d_6_, δ): 24.5 (CH_2_), 44.1 (CH_3_), 106.6, 113.5, 126.2, 127.7, 130.1, 140.4 (C=N), 144.2, 146.0, 150.1, 152.4, 153.6, and 156.1; EI MS *m/z* 323 [M]^+^ (19). Anal. C_16_H_13_N_5_OS: C, 59.43%; H, 4.05%; N, 21.66%; S, 9.92%; Calcd. for C, 59.49%; H, 4.00%; N, 21.64%; S, 9.96%. 

*4-(4-Methyl-4,7-dihydro-[1,2,3]thiadiazolo [4,5-c]pyridazin-6-yl)-N-(thiophen-2-yl methylene)aniline (9i):* Yield: 66%, mp 82–83 °C; IR (KBr) *v_max_* 1626 (C=N) cm^−1^; ^1^H-NMR (DMSO-d_6_, *δ*): 2.84(s, 2H, CH_2_ pyridazine moiety), 3.11 (s, 3H, CH_3_), 6.95–7.86 (m, 7H, Ar–H), 8.84 (s, H, CH); ^13^C-NMR (DMSO-d_6_, δ): 28.0 (CH_2_), 47.9 (CH_3_), 119.2, 125.1, 128.0, 129.2, 133.1, 137.0, 143.2, 146.8, 149.7, 151.0, and 153.7 (C=N), 156.2; Anal. C_16_H_13_N_5_S_2_: C, 56.61%; H, 3.86%; N, 20.63%; S, 18.89 %; calculated. for C, 56.58%; H, 3.83%; N, 20.67%; S, 18.90%.

*General procedure for the synthesis of the thia/selenadiazolopyridazine derivatives 10 and 11:* A mixture of **4** and/or **5** (0.01 mol) and acetonylacetone (0.01 mol) in 30 mL of absolute methanol containing a catalytic amount of glacial acetic acid was refluxed for 15 h, and the resulting solution was concentrated and left to cool. The precipitate formed was filtered, washed with ethanol, dried and recrystallized from ethanol to give compounds **10** and **11**, respectively. 

*6-(4-(2,5-dimethyl-1H-pyrrol-1-yl)phenyl)-4-methyl-4,7-dihydro-[1,2,3]selenadiazolo [4,5-c]pyridazine (10):* Yield: 73%, mp 204–205 °C; IR (KBr) *v_max_* 1620 (C=N) cm^−1^; ^1^H-NMR (DMSO-d_6_, *δ*): 2.86(s, 2H, CH_2_ pyridazine moiety), 1.88 (s, 6H, 2CH_3_ of the pyrrole moiety), 3.13 (s, 3H, CH_3_), 5.22(s, 1H, CH pyrrole moiety), 6.99–7.80 (m, 4H, Ar–H); Anal. C_17_H_17_N_5_Se: C, 55.14%; H, 4.63%; N, 18.91%; Calcd. for C, 55.16%; H, 4.60%; N, 18.88%.

*6-(4-(2,5-dimethyl-1H-pyrrol-1-yl)phenyl)-4-methyl-4,7-dihydro-[1,2,3]thiadiazolo [4,5-c]pyridazine (11):* Yield: 78%, mp 221–222 °C; IR (KBr) *v_max_* 1625 (C=N) cm^−1^; ^1^H-NMR (DMSO-d_6_, *δ*): 2.80(s, 2H, CH_2_ pyridazine moiety), 1.96 (s, 6H, 2CH_3_ of pyrrole moiety), 3.17 (s, 3H, CH_3_), 5.34(s, 1H, CH pyrrole moiety), 7.02–7.89 (m, 4H, Ar–H); EI MS *m/z* 323 [M]^+^ (33). Anal. C_17_H_17_N_5_S: C, 63.13%; H, 5.30%; N, 21.65%; S, 9.91%; Calcd. for C, 63.19%; H, 5.31%; N, 21.60%; S, 9.94%.

### 4.2. Characterization

The melting points were measured on the Electrothermal 9100 melting point apparatus from Electrothermal Engineering Ltd. (Rochford, England). Fourier transform infrared (FTIR) spectra (KBr) were recorded on an FTIR NEXCES spectrophotometer from CTech (Shimadzu, Japan). The 1H NMR spectra were measured with a Jeol ECA 500 MHz instrument (Japan) using deuterated DMSO-*d*6, and chemical changes were recorded in *δ* ppm relative to TMS. Mass spectra (EI) were run at 70 eV with a Finnigan SSQ 7000 spectrometer. The purity of the compounds was obtained using aluminum plates coated with silica gel (Merck). The elemental analysis (C, H, N, and S) was measured using the Costech model 4010, and the percentage values agreed with the proposed structures within ± 0.4 % of the theoretical values.

Antimicrobial Activity: Antimicrobial activities of some of the synthesized compounds were measured for their antibacterial activity against four strains of bacteria, Bacillus subtilis, Staphylococcus epidermidis, Staphylococcus aureus, Salmonella Typhi, and Escherichia coli, and two strains of fungal, namely Aspergillus niger and Candida albicans, using agar disc of nutrients [16,18,25] with a concentration of 100 mg/ml. Dimethylsulphoxide, as a blank, exhibited no activity against any of the organisms used. Antimicrobial activity was evaluated by measuring the inhibition zone after 20–24 h of incubation at 37 °C for bacterial strains and 3–4 days at 37 °C for fungal strains. Ketoconazole and tetracycline were used as reference drugs at concentrations of 30 mg/ml. 

Cytotoxicity Activity: The brine shrimp lethality bioassay [2,40] is the fast and comprehensive bioassay to assess natural and synthetic bioactive compounds, indicating cytocompatibility and a wide range of pharmacological properties. In this technique, the brine shrimp eggs are obtained from the New Aqua Laboratory in Naawan, Misamis Oriental, as a gift. Artificial seawater is prepared by dissolving 38 g of sea salt in 1 L of distilled H_2_O to hatch shrimp eggs and kept in a small tank [2]. The eggs were then added to the divided tank. A constant oxygen supply was provided, and a temperature of 37 °C was maintained for 48 h to hatch and mature the shrimp called nauplii (Larvae). Solutions of compounds 4,5,8a-I and 9a-f were prepared by dissolving 10 mg of each compound in 2 mL of DMSO. From this stock, a series of 5, 10, 20, 40 and 80 mg/mL solutions were transferred to 15 vials (three for each dilution were used for each test sample and LC50 is the mean of three values ± sd), and one vial was kept as a control having 2 mL of DMSO [2]. Then, approximately 10 brine shrimp nauplii are applied to all experimental and control vials. The number of nauplii that died after 24 h was enumerated [2]. The resulting data were transformed to Probit analysis 34 to determine the LC50 values for the four compounds and their derivatives were tested. 

Computational method: All molecular calculations of the optimized structures were performed in the gas phase using density functional theory (DFT) combined with the hybrid functional B3LYP [41]. The calculations were carried out with a base set of 6–31 + G (d) using the Gaussian 16 software package [40]. The fully optimized geometries were proved to be global minima since no imaginary frequencies were found due to calculations in normal mode. Afterward. With the fully optimized geometry structure. The HOMO and LUMO level energy, the LUMO-HOMO frontier orbital gap, and the MESP map were obtained.

## 5. Conclusions

Here we report on the successful synthesis of some new 4,7-dihydro-1,2,3-selena/thiadiazol [4,5-*c*]pyridazine derivatives. Most of the newly synthesized compounds were tested for their antimicrobial activity. The antimicrobial activity study revealed that all the compounds tested showed moderate to high antibacterial and antifungal activities against pathogenic strains. The cytotoxicity of the compounds was also studied, and compounds 8a, 8b, 8e, 8h, and 9a showed the lowest cytotoxicity against brine shrimp lethality bioassay. The theoretical study detailing the optimized structure of different studied structures using DFT calculation is presented. On the one hand, the Frontier molecular orbital (FMO) was studied by calculating the HOMO and LUMO energy levels for evaluating the chemical reactivity and kinetic stability of the molecule. Second-hand. The global descriptor parameters like electronegativity (χ). Chemical hardness (η) and global softness (σ) were calculated. Consequently, we have demonstrated that the structural elements are essential for the chemical reactivity and kinetic stability of each component. A good agreement is established between the experiment and the theoretical calculation, which proves that structure 8c has the highest chemical reactivity and lowest kinetic stability compared to other studied samples.

## Figures and Tables

**Table 1:** Antimicrobial activity of compounds **4**, **5** and **8**–**11**.

Compounds	Minimum Inhibitory Concentration (MIC) in μg/mL					
	Bacterial Strains				Fungal Strains	
	*S. aureus*	*B. subtilis*	*S. typhi*	*E. coli*	*A. niger*	*C. albican*
**4**	50	50	25	50	100	50
**5**	-	100	50	200	-	100
**8a**	25	25	25	12.5	25	25
**8b**	12.5	12.5	12.5	25	12.5	12.5
**8c**	12.5	12.5	25	25	25	50
**8d**	25	25	12.5	12.5	50	25
**8e**	12.5	12.5	12.5	25	12.5	25
**8f**	25	25	25	12.5	50	25
**8g**	12.5	25	50	25	50	50
**8h**	25	50	25	50	50	25
**8i**	12.5	12.5	12.5	25	12.5	25
**9a**	50	50	50	100	100	50
**9b**	25	25	12.5	25	50	25
**9c**	25	50	25	100	25	50
**9d**	100	100	50	100	50	100
**9e**	50	25	50	25	50	50
**9f**	25	50	50	25	100	50
**9g**	25	100	50	50	100	100
**9h**	50	200	100	50	200	50
**9i**	25	25	12.5	12.5	12.5	25
**10**	50	100	100	100	50	200
**11**	50	50	50	100	100	100
Amoxicillin	6.25	6.25	6.25	6.25	-	-
Ketoconazole	-	-	-	-	31.25	31.25

**Table 2:** In vitro cytotoxicity of compounds **4**, **5**, **8**, and **9**.

Compound	IC_50_ (μg/mL) ^a^	Compound	IC_50_ (μg/mL) ^a^
**4**	114 ± 6	**8h**	260 ± 9
**5**	85 ± 4	**8i**	73 ± 12
**8a**	262 ± 9	**9a**	117 ± 45
**8b**	117 ± 71	**9b**	73 ± 12
**8c**	133 ± 11	**9c**	85 ± 5
**8d**	81 ± 3	**9d**	92 ± 18
**8e**	330 ± 45	**9e**	118 ± 7
**8f**	135 ± 11	**9f**	74 ± 11
**8g**	133 ± 25		

^a^ Cytotoxicity of the compounds in the brine shrimp lethality bioassay. Incubation for 24 h. IC_50_ is the drug concentration required to inhibit 50 % of cell growth. The values represent the mean (±) standard deviation of the determinations in triplicate.

**Table 3:** HOMO energy. LUMO energy. and LUMO-HOMO energy gap of compounds calculated using DFT(B3LYP)/6-31G+(d).

Compound Number	HOMO	LUMO	Energy Gap (LUMO-HOMO) (eV)
**1**	−6.25	−1.70	4.54
**2**	−5.58	−1.44	4.13
**3**	−5.48	−1.44	4.03
**4**	−5.33	−1.99	3.34
**5**	−5.47	−1.84	3.63
**6**	−5.08	−1.27	3.80
**7**	−5.59	−2.73	2.85
**8a**	−5.60	−2.24	3.35
**8b**	−5.67	−2.37	3.29
**8c**	−5.84	−3.27	2.56
**8d**	−5.50	−2.15	3.35
**8e**	−5.48	−2.24	3.24
**8f**	−5.50	−2.13	3.37
**8g**	−5.57	−2.49	3.07
**8h**	−5.55	−2.23	3.32
**8i**	−5.57	−2.31	3.26
**9a**	−5.74	−2.21	3.53
**9b**	−5.81	−2.37	3.44
**9c**	−6.00	−3.30	2.70
**9d**	−5.62	−2.07	3.54
**9e**	−5.58	−2.25	3.32
**9f**	−5.63	−2.05	3.58
**9g**	−5.71	−2.51	3.20
**9h**	−5.69	−2.20	3.48
**9i**	−5.71	−2.31	3.40
**10**	−5.35	−2.27	3.08
**11**	−5.38	−2.13	3.25

**Table 4:** Predicted molecular properties of the compounds studied using the DFT method. B3LYP model.6-31G+ basis set.

Compound Number	I (eV)	A (eV)	χ (eV)	η (eV)	ω (eV)	σ (eV)
**1**	6.25	1.70	3.97	2.27	3.48	0.43
**2**	5.58	1.44	3.51	2.06	2.98	0.48
**3**	5.48	1.44	3.46	2.01	2.97	0.49
**4**	5.33	1.99	3.66	1.67	4.01	0.59
**5**	5.47	1.84	3.65	1.81	3.68	0.55
**6**	5.08	1.27	3.18	1.90	2.65	0.52
**7**	5.59	2.73	4.16	1.42	6.08	0.70
**8a**	5.60	2.24	3.92	1.67	4.58	0.59
**8b**	5.67	2.37	4.02	1.64	4.90	0.60
**8c**	5.84	3.27	4.56	1.28	8.10	0.77
**8d**	5.50	2.15	3.82	1.67	4.36	0.59
**8e**	5.48	2.24	3.86	1.62	4.61	0.61
**8f**	5.50	2.13	3.81	1.68	4.32	0.59
**8g**	5.57	2.49	4.03	1.53	5.29	0.65
**8h**	5.55	2.23	3.89	1.66	4.57	0.60
**8i**	5.57	2.31	3.94	1.63	4.76	0.61
**9a**	5.74	2.21	3.98	1.76	4.48	0.56
**9b**	5.81	2.37	4.09	1.72	4.86	0.58
**9c**	6.00	3.30	4.65	1.35	8.01	0.74
**9d**	5.62	2.07	3.85	1.77	4.18	0.56
**9e**	5.58	2.25	3.92	1.66	4.62	0.60
**9f**	5.63	2.05	3.84	1.79	4.11	0.55
**9g**	5.71	2.51	4.11	1.60	5.28	0.62
**9h**	5.69	2.20	3.95	1.74	4.48	0.57
**9i**	5.71	2.31	4.01	1.70	4.72	0.58
**10**	5.35	2.27	3.81	1.54	4.71	0.64
**11**	5.38	2.13	3.76	1.62	4.34	0.61

## Data Availability

Most of the data presented in this study are available in the Supplementary Material. Additional data presented in this study are available on request from the corresponding author.

## Supplementary Materials

The following supporting information can be downloaded at: https://www.mdpi.com/article/10.3390/molecules28031280/s1: Table S1: 3D plots of the molecular orbitals of HOMO and LUMO compounds calculated using the DFT (B3LYP)/6-31G+(d) method.
