# Supplementary material for: 2-Methyl-6-(4-aminophenyl)-4,5-dihydro-3(2H)-pyridazinone Synthon for Some New Annelated 1,2,3-Selena/Thiadiazoles and 2H-Diazaphospholes with Anticipated Biological Activity and Quantum Chemical Calculations
Source: Molecules. 2023 Jan 28;28(3):1280. doi: 10.3390/molecules28031280 (PMC9920368; doi:10.3390/molecules28031280)
Supplement: Supplementary file 1 [file molecules-28-01280-s001.zip › molecules-2172119-supplementary.pdf]

Supporting information

2-Methyl-6-(4-aminophenyl)-4,5-dihydro-3(2*H*)-pyridazinone synthon for some new annelated 1,2,3-selena/thiadiazoles and 2*H*-diazaphospholes with anticipated biological activity and quantum chemical calculations

I. E. El-Shamy [1], E. Hleli[2], M. A. El-Hashash[3], I. Kelnar[2], A. M. Abdel-Mohsen[2*]

[1] Chemistry Department, Faculty of Science, Fayoum University, Fayoum 63514, Egypt

[2]Institute of Macromolecular Chemistry, Czech Academy of Sciences, Heyrovského nám. 2, Praha, 162 06, Czechia

[3]Chemistry Department, Faculty of Science, Ain Shams University, Cairo, Egypt.

Corresponding author at:

E-mail addresses: abdellatif@imc.cas.cz , (A.M. Abdel-Mohsen)

Table S1: 3D plots of HOMO and LUMO molecular orbitals of compounds calculated using DFT(B3LYP)/6-31G+(d) method.

	HOMO	LUMO
8b	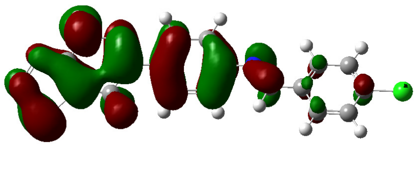	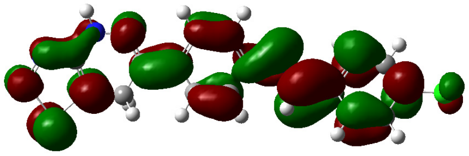
8e	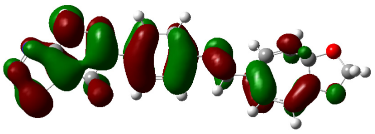	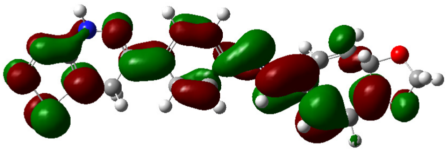
8f	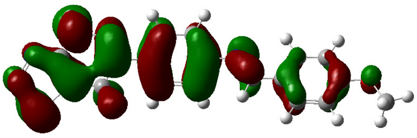	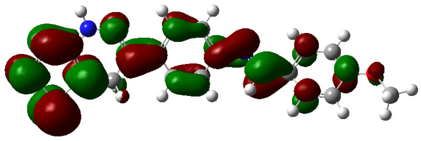
8g	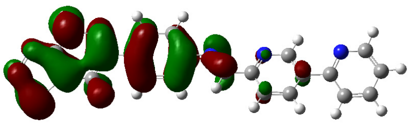	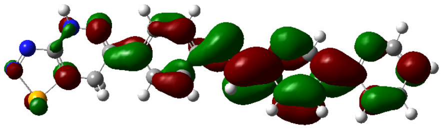
8h	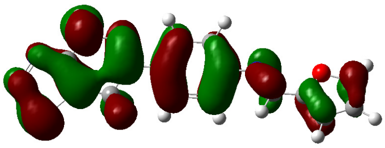	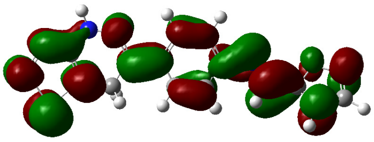
8i	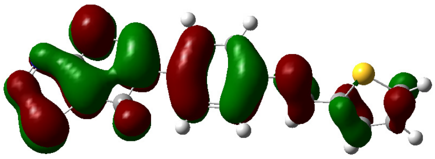	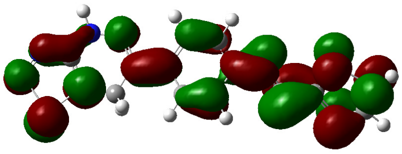
9a	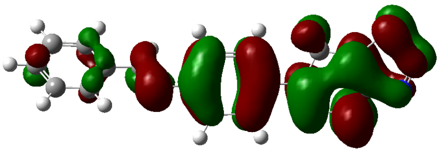	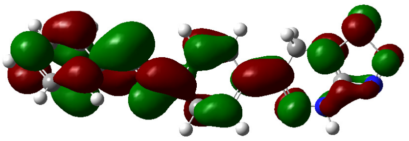
9b	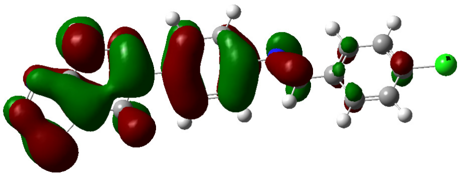	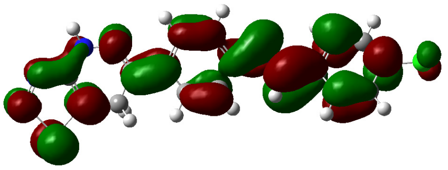

9e	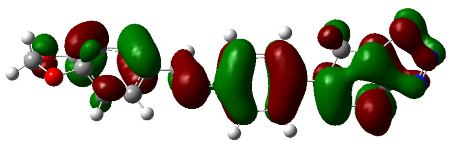	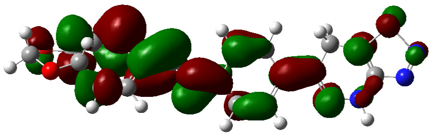
9f	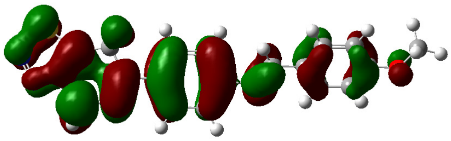	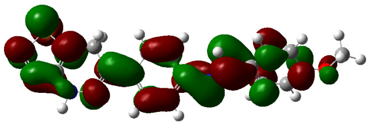
9g	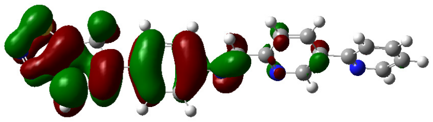	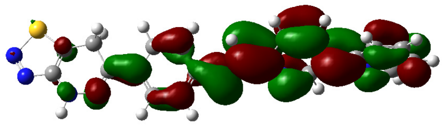
9h	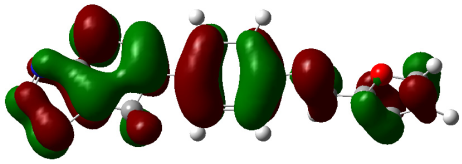	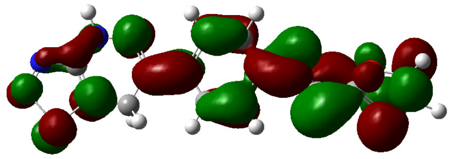
9i	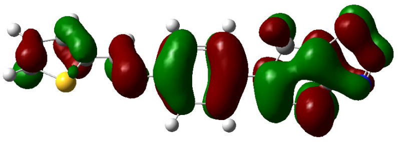	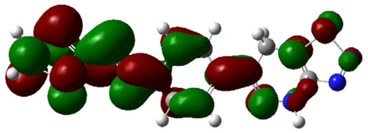
